# A thank you to all our reviewers

**DOI:** 10.1308/rcsann.2025.0126

**Published:** 2025-01-01

We would like to take this opportunity to thank all our reviewers for the time and expertise you generously contribute to peer-reviewing articles submitted to the *Annals of the Royal College of Surgeons of England*. We want to acknowledge your support during 2025, without which it would not be possible to preserve the high standards of our journal.

Abdalla, Leena
Abdallah, Mohamed
Acheson, Austin
Afrooz, Selina
Ahmed, Shakil
Al-Mothenna, Alloush
Ananth, Sankar
Angamuthu, Natarajan
Ashwood, Neil
Asif, Aqua
Baker, Daryll
Balamurali, Bharathan
Baldwin, Alexander
Banham, Evie
Bater, Michael
Batten, Timothy
Battersby, Chris
Bedford, James
Bhardwaj, Rakesh
Bhargava, Eishaan K.
Bhatt, Yogesh
Bhattacharya, Satya
Bhutta, Mahmood
Bonomaully, Matthew
Booker, Simon
Borley, Neil
Bowbrick, Ginny
Bowrey, David
Boyle, Kirsten
Brewin, James
Britton, David
Buckle, Christopher
Burden, Eleanor
Burns, Elaine
Busuttil, Andrew
Butt, Muhammad
Bylapudi, Seshu
Cahill, Ronan
Canu, Gian Luigi
Carter, Alison
Chan, David
Chan, Gareth
Chandran, Suresh
Chaturvedi, Ankita
Chircop, Daniel
Chow, James C. L.
Chui, Karen
Cimprich, Alexander
Clifford, Rachael
Coleman, Mark
Cook, Tim
Costello, Declan
Coyle, Paula
Cripps, Neil
Cromwell, David
Dangerfield, Peter
Davenport, Mark
Day, Andrew
de Joseph, Jomon
Desai, Mital
Dickson, Edward
Dinets, Andrii
Doran, Helen
Dream, Sophie Y.
Drebes, Anja
D'Souza, Rovan
Dunlop, Doug
Eastwood, Deborah
El-Sharkawy, Ahmed
Emara, Khaled
English, William
Estefan, Martin
Evans, Julian
Eyres, Keith
Ezzat, Ahmed
Fang, Chien-Liang
Faure Walker, Nicholas
Fleming, Myles
Fleming, Simon
Fries, C
Gaba, Swati
Ganau, Mario
Gani, Jon
Geh, Jenny
George, Manish
Giannoudis, Peter
Gillies, Martin
Glover, Anthony
Griffiths, Ewen
Hamish, Maher
Hampton, Thomas
Hance, Julian
Hancock, Laura
Hardcastle, Timothy
Hardie, John
Harji, Deena
Harris, Anna
Harris, Grant
Hill, Sue
Hill, Susan
Holt, S
Howard, Matthew
Hubbard, Thomas
Hussain, Zahra
Ikeda, Atsushi
Inston, Nicholas
Iype, Satheesh
Jones, Oliver
Joshi, Heman
Kadri, Sudarshan
Karuppaiah, Karthik
Karwasra, Isha
Kavia, Rajesh
Kelly, Mike
Kenington, Cleo
Keshishian, Karekin
Khong, Grace
Kulkarni, Kunal
Lamyman, Abigail
Lau, Andrew
Lee, Matthew
Lim, Chung
Litsou, Eleni
Loan, James J. M.
Lockyer, Richard
Loizou, Peter
Lovegrove, Catherine
Lovegrove, Richard
Lund, Jonathan
Luther, Alison
Machesney, Michael
Macklin, Chris
Mactier, Mhairi
Magee, Conor
Martin, Lee
Martin, Sean
McAleer, Joe
McDermott, Frank
McLaughlin, Joanna
Metcalfe, Christopher
Mihai, Radu
Milinis, Kristijonas
Miller, Andrew
Millwaters, Michael
Mirshekar-Syahkal, Bahar
Mirza, Yusuf
Mishra, Ami
Motawea, Mahmoud
Mott, Andrew
Moug, Susan
Mullasserry, Dhanya
Musbahi, A
Mutimer, Jonathan
Nadarajah, Vicki
Nastos, Konstantinos
Navsaria, Pradeep
Neri, Giampiero
Ng, Jessica
Nickel, Felix
Nour, Hussam
Oddy, Mike
Olyslaegers, Christophe
Osei-Nketiah, Samuel
Osman Farah, Jibril
Paranathala, Menaka
Patel, Vipul
Paul, Melanie
Phelan, Liam
Plock, Jan A.
Probst, Pascal
Pucher, Philip
Puttergill, Brooke
Quiroga, Isabel
Ramsay, George
Repo, Jussi P.
Reynolds, Patrick
Richards, Simon J. G.
Robertson, Andrew
Rockall, Tim
Roy, Holly
Rumi, Shaikh Nurul Fattah
Rutenberg, Tal Frenkel
Saad, Alaaeldin
Saleh, Raoof
Samant, Ambaresh
Sandhu, Guri
Sargazi, Nastaran
Saunders, Rebecca
Senapati, A
Sharma, Neil
Silickas, Justinas
Singh, Anjana
Singh, Dr DP
Sivaraj, Jayaram
Solan, Matthew
Soundy, Andrew
Sritharan, Kaji
Tan, Jih Huei
Tatterton, Mark
Tekkis, Paris
Thomas, William
Tierney, Gillian
Tiwari, Alok
Totty, Joshua
Trickett, Ryan W.
Uppal, Harpal
Vimalachandran, Dale
Visavadia, Bhavin
Walsh, Ciaran
Wernig, Florian
Wheeler, James
Wijetunga, Imeshi
Williams, Gethin
Yardley, Iain
Zamora, Bernarda
Zeiton, Moez

